# The technology acceptance model and adopter type analysis in the context of artificial intelligence

**DOI:** 10.3389/frai.2024.1496518

**Published:** 2025-01-16

**Authors:** Fabio Ibrahim, Johann-Christoph Münscher, Monika Daseking, Nils-Torge Telle

**Affiliations:** ^1^Faculty of Humanities and Social Sciences, Helmut-Schmidt-University/University of the Armed Forces, Hamburg, Germany; ^2^Independent Researcher, Hamburg, Germany

**Keywords:** artificial Intelligence, technology acceptance model, big five, AI mindset, early adopter, late adopter

## Abstract

**Introduction:**

Artificial Intelligence (AI) is a transformative technology impacting various sectors of society and the economy. Understanding the factors influencing AI adoption is critical for both research and practice. This study focuses on two key objectives: (1) validating an extended version of the Technology Acceptance Model (TAM) in the context of AI by integrating the Big Five personality traits and AI mindset, and (2) conducting an exploratory k-prototype analysis to classify AI adopters based on demographics, AI-related attitudes, and usage patterns.

**Methods:**

A sample of *N* = 1,007 individuals individuals (60% female; *M* = 30.92; SD = 8.63 years) was collected. Psychometric data were obtained using validated scales for TAM constructs, Big Five personality traits, and AI mindset. Regression analysis was used to validate TAM, and a k-prototype clustering algorithm was applied to classify participants into adopter categories.

**Results:**

The psychometric analysis confirmed the validity of the extended TAM. Perceived usefulness was the strongest predictor of attitudes towards AI usage (*β* = 0.34, *p* < 0.001), followed by AI mindset scale growth (*β* = 0.28, *p* < 0.001). Additionally, openness was positively associated with perceived ease of use (*β* = 0.15, *p* < 0.001). The k-prototype analysis revealed four distinct adopter clusters, consistent with the diffusion of innovations model: early adopters (*n* = 218), early majority (*n* = 331), late majority (*n* = 293), and laggards (*n* = 165).

**Discussion:**

The findings highlight the importance of perceived usefulness and AI mindset in shaping attitudes toward AI adoption. The clustering results provide a nuanced understanding of AI adopter types, aligning with established innovation diffusion theories. Implications for AI deployment strategies, policy-making, and future research directions are discussed.

## 1 Introduction

Artificial Intelligence (AI) describes a computer technology with “human-like thought processes such as learning, reasoning, and self-correction” (Dilsizian and Siegel, 2013). The autoregressive large language model GPT-3 (Generative Pre-trained Transformer) and its current successor GPT-4 are computational systems that generate word or data sequences based on an initial user input (prompt) and even demonstrate various features of intelligence (Floridi and Chiriatti, 2020). Notably, with the GPT-4 model as its underlying technology, ChatGPT is considered a tipping point for AI, showcasing the possibilities of this new technology to the general public (Doshi et al., 2023). AI has become a transformative tool in numerous industries and is seen as a means to enhance human capabilities at low cost (Schwab, 2017). AI is regarded as a key success factor for future industries making AI employee recruitment and expertise increasingly important in the field of human resources to achieve a symbiosis between AI and the workforce (Jarrahi, 2018). Policymakers, managers, and society should prioritize enhancing rather than replacing the human workforce. Pursuing a human-complementary approach could foster economic growth and promote greater economic equality (Capraro et al., 2024). To improve the symbiotic relationship in the context of human-AI interaction, the investigation and optimization of AI design approaches alone are not sufficient, as attitudes towards AI usage vary individually (Vasiljeva et al., 2021). Therefore, it will be important for companies and organizations to measure and proactively change attitudes towards AI, as this acceptance significantly influences behavioral intention, as described in the Technology Acceptance Model.

## 2 Theoretical background

### 2.1 The technology acceptance model in the context of AI

The Technology Acceptance Model (TAM) (Davis, 1989) is considered the most widespread model for measuring user acceptance and describes the behavioral intention or willingness to use a technology through underlying attitudes (Kelly et al., 2023). The model includes the predictors perceived usefulness, defined as the belief that the technology use enhances performance, and perceived ease of use, defined as the belief that its use is free of effort (Davis, 1989). Both predictors influence the attitude towards use, which in turn affects the behavioral intention to use.

The model was extended by Venkatesh and Davis (2000) to include additional cognitive constructs, such as subjective norm, referring to the influence of peer or supervisory pressure and positive identification with the technology on technology acceptance. As companies increasingly aim to enhance employee productivity through the use of AI (Østerlund et al., 2021) and adopt this technology, subjective norms emerge as a crucial predictor in the investigation of AI technology acceptance.

The TAM allows for the adaptation of items to the specific technology, and thus has been used to measure the acceptance of AI technology. Studies show that perceived ease of use and perceived usefulness also impact attitude towards use in the context of AI use (Sohn and Kwon, 2020; Wang et al., 2023; Zou and Huang, 2023). In the study by Zou and Huang (2023), attitude towards use is identified as an important factor influencing behavioral intention. However, in the study by Sohn and Kwon (2020), which examined AI consumer acceptance, attitude towards use did not influence behavioral intention; only perceived ease of use and subjective norm were relevant predictors of behavioral intention. Similarly, in Wang et al. (2023), where AI was studied in the context of e-commerce, attitude towards use did not affect behavioral intention, and subjective norm was found to influence perceived usefulness and perceived ease of use. In this study, we replicate the TAM from Zou and Huang (2023), including subjective norm as a predictor of attitude towards use. Additionally, we extend the model by incorporating the proximal predictor of AI mindset.

### 2.2 The technology acceptance model extended by personality

An important predictor considered in the study of technology acceptance and specifically within the TAM framework as an additional influencing factor is personality (Devaraj et al., 2008). One of the most central and well-validated personality taxonomies is the Big Five, which forms a hierarchical model of five global traits: openness, conscientiousness, extraversion, agreeableness, and neuroticism (Costa and McCrae, 2008). The relationship between the Big Five and TAM has been explored in numerous studies. In the study of technology acceptance of digital applications for data management by Svendsen et al. (2013), it was found that conscientiousness positively influenced subjective norm. Emotional stability showed a positive influence on perceived ease of use. Openness and extraversion positively influenced perceived ease of use, with extraversion also positively affecting perceived usefulness. In the study by Devaraj et al. (2008), agreeableness was additionally found to be positively associated with perceived usefulness. However, the influence of personality on TAM in the context of AI usage remains a relatively unexplored area, despite the context specificity being of central importance in TAM research (McFarland and Hamilton, 2006).

Furthermore, the study by Schepman and Rodway (2023) demonstrated that the general attitude towards artificial intelligence is predicted by extraversion. Introverts tend to have a more positive attitude towards AI, which is also supported by the results on algorithm appreciation (Logg et al., 2019). Research on the construct of mindset shows that openness predicts growth mindset (Billingsley et al., 2023), suggesting that higher levels of openness are also associated with AI mindset, particularly the growth mindset subscale.

### 2.3 The incremental belief towards AI—the AI mindset

The way in which a new subject matter is engaged and subsequently pursued is often shaped by theories or beliefs. They are general assumptions which are not necessarily explicitly expressed or derived from evidence or experience such as attitudes. With regards to such beliefs, two major outlooks can be differentiated: Incremental beliefs assume a generally flexible situation in which growth can be facilitated whereas entity beliefs assume that outcomes are determined by a mostly static situation. Extending this idea, the Mindset theory assumes two beliefs that individuals can hold: the growth mindset is characterized by an optimistic outlook in which growth is attainable through personal development (an incremental belief). The fixed mindset on the other hand is characterized by a somewhat pessimistic outlook in which the individual cannot enact a positive influence and is even subject to detriments. Previous research indicates that mindset is vital in many fields of performance and achievement. Holding a growth mindset was often shown to be advantageous for performance and success (Dweck and Yeager, 2019).

An early application of the mindset theory in the context of AI was presented by Chaffee (1991) in which research on attitudes towards AI was aggregated. However, mindset and attitude are not the same. Attitudes are generally directed at a subject (distal), while mindset constitutes beliefs about oneself (proximal) in the context of a subject. Thus, a recent approach by Ibrahim et al.^1^ refined the approach by constructing a theoretical model of an AI mindset comprising two dimensions: growth and non-deskilling. In this model, growth describes the belief that AI expands the individuals’ capability while non-deskilling is characterized by the belief that using AI does not impede the individual’s abilities. Despite the findings on the construct validity of the AI mindset, there are currently no results regarding the possible incremental validity within the technology acceptance model.

### 2.4 Artificial intelligence user types

The implementation of AI tools as disruptive technologies significantly affects (organizational) adoption patterns, as outlined in Rogers’ Diffusion of Innovations Theory. Rogers categorizes adopters into innovators, early adopters, early majority, late majority, and laggards (Rogers, 2003). Innovators (2.5%) are the first to adopt AI, driven by a passion for cutting-edge technology and risk tolerance. Early adopters (13.5%), such as industry leaders, follow, leveraging AI for competitive advantages (Venkatesh et al., 2003). The early majority (34%) adopts AI after its benefits are validated by early adopters, aiming to enhance efficiency and productivity (Damanpour and Schneider, 2006). The late majority (34%) is more cautious, requiring substantial evidence and peer validation before integrating AI into their operations (Rogers, 2003). Finally, laggards (16%) resist adopting AI until it becomes unavoidable or industry-standard (Cascio and Montealegre, 2016).

To gain a deeper understanding of different groups adopting new AI technology, investigating demographic as well as personality factors seems promising to begin with. Initial research shows that males hold a more positive attitude and acceptance toward AI compared to females (e.g., Fietta et al., 2022; Schepman and Rodway, 2023; Sindermann et al., 2022) although some researchers report no gender differences (Kaya et al., 2024). Similarly, there is conflicting research evidence regarding age. While research done by Kaya et al. (2024) and Chocarro et al. (2021) point into the direction that age does not predict attitudes toward AI or the likelihood of adopting such technology, other research does (e.g., Park and Woo, 2022).

Investigating which psychological and personality factors predict attitudes toward AI and its usage, considerable research has been done to (e.g., Kaya et al., 2024; Nadarzynski et al., 2019; Park and Woo, 2022). However, to date, research in the AI context that describes Rogers’s (2003) different adoption groups with specific psychological traits is still scarce. Regarding technological innovation in general, Tverskoi et al. (2022) characterize early adopters as individuals with low cognitive dissonance and low felt pressure to conform with their peers. At the same time, they are described to be sensitive to activities targeted to promote the technological innovation by an external authority. Focusing on AI technology, Haque et al. (2022) investigated sentiments of ChatGPT early adopters. With ChatGPT having gained great popularity among early adopters the sentiments were—unsurprisingly—largely positive and revolving around the topics of disruption to software development, entertainment and creativity. Haque and colleagues found only few negative sentiments in their field-data. These negative sentiments included topics like the potential misuse of ChatGPT or the negative impact on education.

With a detailed description of AI adoption groups being largely absent, we consider it worthwhile to start illuminating this field. By aligning AI tools with user traits, capabilities and expectations, companies and organizations can enhance user engagement, reduce resistance and optimize the integration process. Such people-centered approach allows for a smoother transition and maximizes the return on investment in AI technologies.

### 2.5 The acceptance and adoption of artificial intelligence—aim of this study

**Figure 1:**
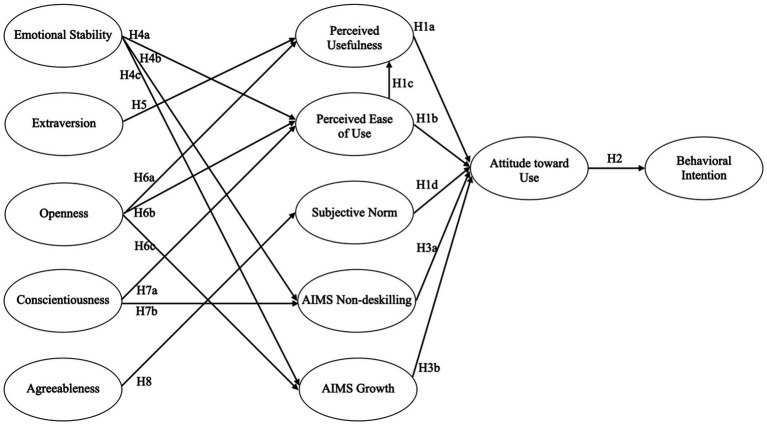
Hypotheses on the expanded technology acceptance model in the context of AI.

Due to the growing importance of AI adoption and acceptance for society and the economy, this study investigates whether the TAM can be replicated in the context of AI. Additionally, we extend the TAM by incorporating the Big Five personality traits and the AI mindset to derive potential predictors of AI-specific technology acceptance. Finally, we exploratively determine whether distinct AI user prototypes can be identified, which may provide insights into AI adoption. The hypotheses are presented in Table 1 and Figure 1.

**Table 1:** Structural model results.

Hypothesis	Path	Path coefficient	*t*	Accepted
TAM				
H1a	PU → ATU	0.34^***^	12.7	Yes
H1b	PEU → ATU	0.22^***^	9.40	Yes
H1c	PEU → PU	0.56^***^	21.30	Yes
H1d	SocNorm → ATU	0.16^***^	7.23	Yes
H2	ATU → BI	0.73^***^	33.85	Yes
AIMS				
H3a	Non-D → ATU	0.03	1.30	No
H3b	Growth → ATU	0.28^***^	10.92	Yes
Big five				
H4a	N → PEU	−0.05	−1.54	No
H4b	N → Non-D	−0.10^***^	−3.13	Yes
H4c	N → Growth	−0.12^***^	−3.71	Yes
H5	E → PU	0.06	2.05	No
H6a	O → PU	0.03	1.22	No
H6b	O → PEU	0.15^***^	21.30	Yes
H6c	O → Growth	0.15^***^	4.81	Yes
H7a	C → PEU	0.08^*^	2.38	No
H7b	C → Non-D	0.15^***^	4.84	Yes
H8	A → SocNorm	0.11^***^	3.76	Yes

OP, openness; PU, perceived usefulness; PENJ, perceived enjoyment; PEU, perceived ease of use; ATU, attitude toward use; BI, behavioral intention; ^*^/^**^/^***^ according to *p* < 0.05/0.01/0.001.

## 3 Method

### 3.1 Procedure

**Figure 2:**
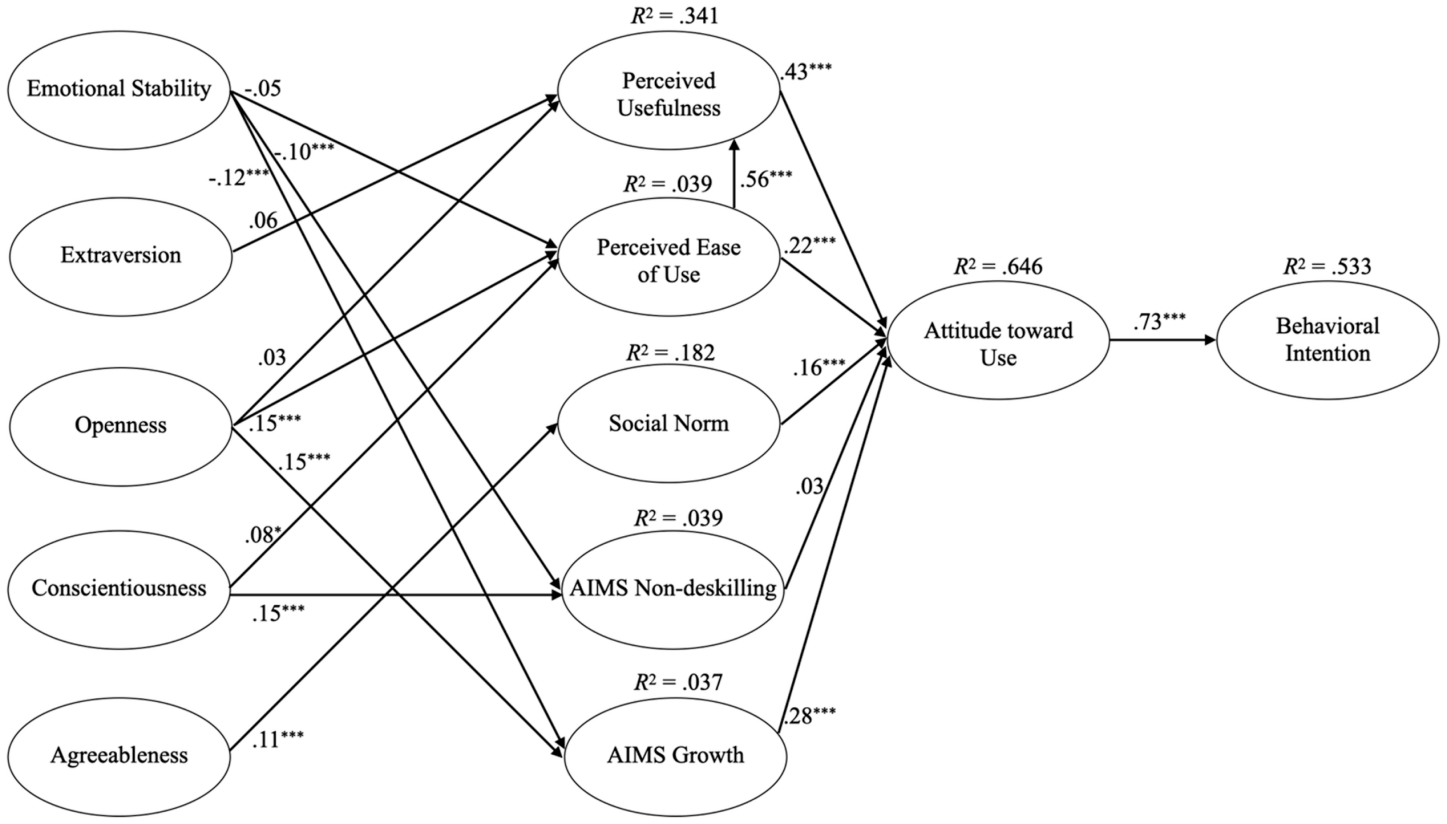
The PLS-SEM of model 1 with standardized path coefficients; ^*^/^**^/^***^ according to *p <* 0.05/0.01/0.001.

The online survey was conducted from October 2023 to February 2024 using the survey platform form{‘r} (Arslan et al., 2020). The generated overall dataset was utilized for two research studies. Sample acquisition was carried out through invitations on the online platforms LinkedIn and Survey Circle. Additionally, a commercial survey panel (Consumerfieldwork GmbH) was commissioned to specifically recruit non-student participants (see Figure 2).

The ethical approval of the study was coordinated with the Chair of the Ethics Committee at the Helmut-Schmidt-University/ University of the German armed forces in Hamburg, Germany and the study was conducted according to the ethical principles of the declaration of Helsinki. Therefore, the data were collected completely anonymously, and all participants consented to data processing and voluntarily participated in the study. A prerequisite for study participation was at least one prior experience with AI. The total sample comprised 1,033 individuals, with 20 observations excluded due to incorrectly answered control scales. An outlier analysis using Mahalanobi’s distance measure identified 52 outliers (*p* < 0.001). However, inspection of these outliers revealed plausible response behavior and consistent open-ended answers. Therefore, we decided not to exclude them to preserve the natural variance of the data. The final sample (*n* = 436 men, 40%) had an average age of 30.92 years (*SD* = 8.63) and mostly held a bachelor’s degree (*n* = 370, 37%), with many in full-time employment (*n* = 482, 48%). Further sample details are provided in Table 2.

**Table 2:** Sample demographics.

	Sample		
	Male	Female	Total
Size (total sample size in %)	436 (40%)	568 (60%)	1,004
Age	33.06 (9.56)	29.29 (7.45)	30.92 (8.63)
Middle school leaving certificate	20 (5)	37 (7)	57 (6)
A-levels	107 (25)	156 (27)	263 (26)
Bachelor’s degree	158 (36)	212 (37)	370 (37)
Master’s degree	131 (30)	144 (25)	275 (27)
Doctorate	20 (5)	19 (3)	39 (4)
Occupational status			
Full-time job	261 (60)	221 (39)	482 (48)
Part-time job	26 (6)	61 (11)	87 (9)
Job seeker	9 (2)	6 (1)	15 (2)
Pensioner	2 (1)	1 (<1)	3 (<1)
Self-employed	13 (3)	14 (3)	27 (3)
Housewife/househusband	0	12 (2)	12 (2)
Student	125 (29)	253 (45)	378 (38)

Standard deviation or percentages in brackets; educational qualifications “no school leaving certificate,” “secondary modern school” were not listed due to a lack of response; *n* = 3 identified as divers were excluded.

### 3.2 Transparency and openness

All data, analysis code, and research materials are available at https://osf.io/uzhsn/?view_only=7e24d9640d1a406f9d1a8d529ac6aa33. Data were analyzed using R, version 4.4.0 (R Core Team, 2024) and the packages *plspm*, version 0.5.1 (Sanchez et al., 2024), *lavaan* (Rosseel, 2012), clustMixType (Szepannek, 2018), and *psych* version 2.4.3. This study’s design and its analysis were preregistered available at https://osf.io/pc5vf/?view_only=a4bca015bbd64f37a1d5f67559831d13.

### 3.3 Instruments

#### 3.3.1 German AI adapted technology acceptance model

Following the AI adaptation by Sohn and Kwon (2020), the German translation consists of a total of 20 items, which form 5 scales. The items were answered on a 5-point Likert scale from 1 (strongly disagree) to 5 (strongly agree). The scales perceived usefulness (PU), perceived ease of use (PEU) Venkatesh and Davis, 2000), behavioral intention (BI), attitude towards use (ATU) (Rahman et al., 2017), and subjective norm (SN) (Venkatesh et al., 2003) demonstrated very good to excellent reliabilities in the study by Sohn and Kwon (*ɑ* = 0.81–0.93).

#### 3.3.2 Big five inventory short scale (BFI-S)

The BFI short (BFI-K) (Rammstedt and John, 2005) comprises 21 items, each rated on a 5-point Likert scale ranging from 1 (very wrong) to 5 (very true). This instrument assesses personality across the dimensions of openness, neuroticism, conscientiousness, extraversion, and agreeableness, exhibiting internal consistency ranging from acceptable to very good (*α* = 0.62–0.87).

#### 3.3.3 The AI mindset scale (AIMS)

The AI Mindset Scale^2^ consists of a total of eight items, which are rated on a 6-point Likert scale ranging from 1 (strongly disagree) to 6 (strongly agree). This instrument includes a total score as well as the sub-scales non-deskilling, reflecting the belief that using AI does not diminish one’s abilities, and growth, reflecting the belief that using AI can aid in one’s potential development. The scales demonstrate very good reliability (*α* = 0.82–0.91).

### 3.4 Statistical analysis

For hypothesis testing, we used partial least squares structural equation modeling (PLS-SEM), which is well-suited for theoretical model extensions, smaller sample sizes, and normality violations (Hair et al., 2011). Unlike covariance-based SEM, PLS-SEM maximizes the explained variance of dependent constructs, making it ideal for predictive modeling and exploratory model extensions. Given our study’s extension of the Technology Acceptance Model (TAM) with personality and mindset constructs, PLS-SEM was chosen for its robustness in estimating path relationships under these conditions. PLS paths were examined using the R software (R Core Team, 2024) along with the plspm package (Esposito Vinzi et al., 2008). To interpret effect sizes, we followed guidelines proposed by Gignac and Szodorai (2016) (*r* = 0.10/0.20/0.30 for small/moderate/large effects) and Sanchez et al. (2015) (R^2^ < 0.30/0.60/>0.60 for small/moderate/large effects).

Incomplete data sets and participants who wrongly answered the control scales (*n* = 20) were excluded. The outlier analysis with Mahalanobi’s distance measure did indicate *n* = 52 outliers (d-squared value, *p* < 0.001), but the data inspection, especially the open answers indicated valid answeres, therefore, we decided to not exclude further cases.

Sample size determination followed the 10-times rule method, commonly employed in PLS-SEM (Hair et al., 2011). The minimum sample size was calculated based on the maximum number of paths to or from a latent variable (Goodhue et al., 2012), yielding *n* = 80 for the model. Additionally, meeting the more conservative minimum sample size requirement using the minimum R-squared method (Hair et al., 2021) recommended a sample size of *n* = 488, considering a power of 0.08, a significance level of <0.05, an expected effect size of 0.2, with 49 observed variables, and 12 latent variables.

Following recommendations by Hair et al. (2022), SEM was examined in two steps. At first we examined the measurement model, evaluating indicator reliability, internal consistency, convergent validity (assessed via average variance explained), and discriminant validity (examined using heterotrait-monotrait ratio). In the second step, the structural model was analyzed to address hypotheses, focusing on path coefficients and explained variance of endogenous constructs.

Moreover, we explored different AI user groups within both the total sample and a subsample of individuals using AI at work. The k-prototype algorithm, implemented via the clustMixType package (Szepannek, 2018), was used to analyze metric and categorical variables simultaneously, assigning observations to their nearest clusters. Determination of the appropriate number of clusters utilized a scree test, with the elbow criterion guiding cluster selection.

Finally, open-ended responses to the question: “Please describe what you use artificial intelligence for at work” underwent qualitative content analysis (Stamann et al., 2016) employing an inductive approach to derive categories from the responses. The open-ended answers were assigned to categories based on keywords, such as “debugging” or “translation.” If a response included multiple aspects, it was counted in several categories.

## 4 Results

### 4.1 Expansion of the technology acceptance model in the context of AI

#### 4.1.1 Measurement model

To investigate the extension of the technology acceptance model in the context of AI, the measurement model and, in the first step, the indicator reliabilities were examined. Here, the conscientiousness item 2 (λ = 0.210), the agreeableness item 2 (*λ* = 0.236), the technology acceptance model subjective norm items 3 and 4 (*λ* = 0.353; *λ* = 0.362), and the behavioral intention item (λ = 0.097) fall below the required loading threshold (*λ* ≥ 0.40; Hair et al., 2019) after two iterations and were, therefore, excluded. Recalculation of the measurement model then showed sufficient loadings (*λ* = 0.424–0.933). The examination of the internal consistency indicated good to very good reliabilities for the Big Five (openness, 𝜌D.G. = 0.859; extraversion, 𝜌D.G. = 0.889; conscientiousness, 𝜌D.G. = 0.863; neuroticism, 𝜌D.G. = 0.875; agreeableness, 𝜌D.G. = 0.803), the AI mindset scale (non-deskilling, 𝜌D.G. = 0.925); growth, (𝜌D.G. = 0.920), the technology acceptance model (perceived ease of use, 𝜌D. G. = 0.883; social norm, 𝜌D.G. = 0.919), perceived usefulness, 𝜌D.G. = 0.944; attitude toward use, 𝜌D.G. = 0.880; behavioral intention, 𝜌D.G. = 0.905). To examine the convergent validity, the AEV was examined, and again, agreeableness was the only construct below the threshold (AVE = 0.498). The other constructs fulfill the validity criteria (AEV = 0.533–0.849). As the quality criteria was nearly reached, we chose not to exclude agreeableness from the model. The HMTM indicated discriminant validity as all correlations fell below the threshold according to Henseler et al. (2015) (HTMT < 0.730).

#### 4.1.2 Structural model and hypothesis testing

The path coefficients of the PLS-SEM were examined to test the hypotheses (Figure 1; Table 1). The explained variance of the endogenous variables thought the model was: perceived ease of use 3.9%, perceived usefulness 34.1%, subjective norm 18.2%, non-deskilling 3.9%, growth 3.7%, attitude toward use 64.6%, and behavioral intention 53.3%. According to the recommendations of Sanchez et al. (2015), the predictive power for behavioral intention was medium and strong for attitude toward use.

The examination of the path coefficients supported the TAM, as perceived usefulness (*β* = 0.34, *p* < 0.001; H1a), perceived ease of use (*β* = 0.22, *p* < 0.001; H1b) and subjective norm (*β* = 0.16, *p* < 0.001; H1d) were associated with attitude toward use (Figure 2). Further, perceived ease of use was strongly correlated with perceived usefulness (*β* = 0.56, *p* < 0.001; H1c). Attitude toward use also strongly correlated with behavioral intention (*β* = 0.73, *p* < 0.001), supporting hypothesis 2. Examining the AIMS effect on attitude toward use indicated no effect of non-deskilling (*β* = 0.03, *p* = 0.223; H3a) and a positive effect of growth (*β* = 0.28, *p* < 0.001), supporting hypothesis 3b. The Big Five’s effect on the TAM indicated no association of neuroticism with perceived ease of use (*β* = −0.05, *p* = 0.123), leading to the rejection of hypothesis 4a, but a negative association with non-deskilling (*β* = −0.10, *p* < 0.001; H4b) and growth (*β* = −0.12, *p* < 0.001; H4c). Extraversion (*β* = 0.06, *p* = 0.087) and Openness (*β* = 0.03, *p* = 0.225) were not associated with perceived usefulness, therefore, hypotheses 5 and 6a were rejected. Yet, openness showed a positive association with perceived ease of use (*β* = 0.15, *p* < 0.001; H6b) and growth (*β* = 0.15, *p* < 0.001; H6c). Conscientiousness showed a significant but weak association with perceived ease of use (*β* = 0.08, *p* = 0.02), therefore rejecting hypothesis 7a, and a positive association with non-deskilling (H7b). Lastly, agreeableness was positively correlated with subjective norm, leading to the acceptance of hypothesis 8.

### 4.2 User adoption type analysis

**Figure 3:**
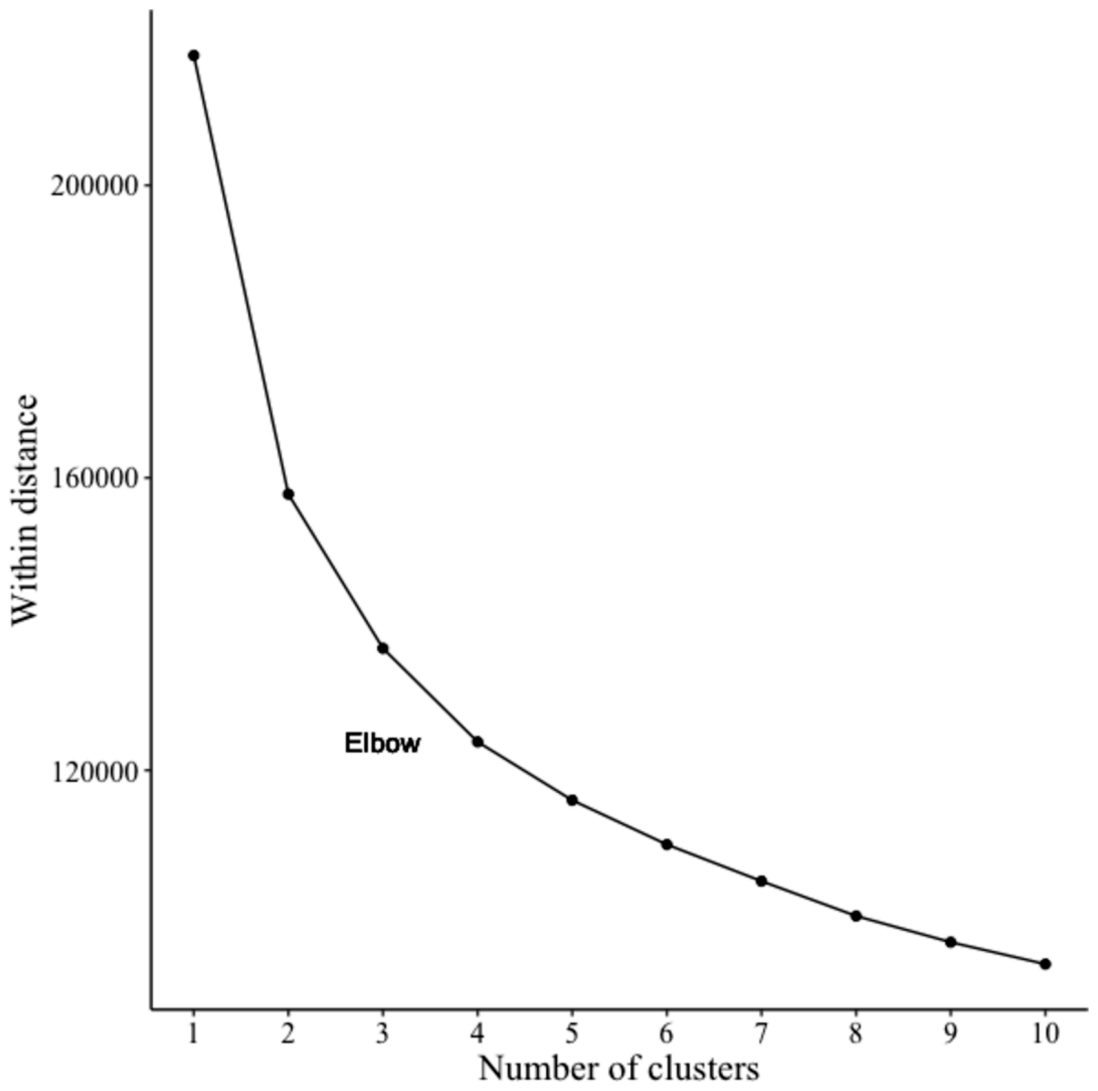
Scree plot of the different k-prototype solutions indicating the weighted distance (withinss).

For the exploratory examination of the total sample (*n* = 1,007) concerning different types in AI adoption, we included four categorical variables (gender, job status, primary AI purpose) and six metric variables (age, AI frequency of use, general computer experience, attitude toward use, non-deskilling, and growth). Determining the number of clusters based on a jump in within distances (elbow criteria; Figure 3) led to the selection of a four-cluster solution as most parsimonious.

The first cluster (*n* = 293) is predominantly female (77%), with a mean age of *M* = 24.09 (*SD* = 3.03) years, primarily consisting of students (*n* = 228; 78%) with A-levels (*n* = 167; 57%), utilizing AI for information purposes (*n* = 159; 54%). The second cluster (*n* = 331) predominantly includes females (*n* = 212 males; 64%), with a mean age of *M* = 32.90 (*SD* = 3.62) years, mostly holding full-time jobs (*n* = 253; 76%) and having a master’s degree (52%), utilizing AI for work (*n* = 160; 48%). The third cluster (*n* = 218) includes 132 males (61%), with a mean age of *M* = 25.58 (*SD* = 3.16) years, mainly consisting of students (*n* = 136; 62%) with a bachelor’s degree (*n* = 127; 58%), utilizing AI for work (*n* = 123; 56%). The fourth cluster (*n* = 165; Table 3) comprises 118 males (72%), with a mean age of *M* = 46.04 (*SD* = 6.44) years, predominantly holding full-time jobs (*n* = 127; 77%) and having a master’s degree (*n* = 77; 47%), utilizing AI for information purposes (*n* = 160; 48%).

**Table 3:** Cluster solution for the total sample.

	Cluster 1	Cluster 2	Cluster 3	Cluster 4
	Early adopter	Early majority	Late majority	Laggard
Size	218 (22%)	331 (33%)	293 (29%)	165 (16)
Male	132 (61%)	119 (36%)	67 (23%)	118 (72%)
Age	25.58 (3.16)	32.90 (3.62)	24.09 (3.03)	46.04 (6.44)
Computer experience	4.22 (0.73)	4.07 (0.779)	3.55 (0.79)	3.96 (0.80)
AI use frequency	4.98 (1.51)	3.73 (2.04)	3.59 (1.78)	3.14 (1.82)
Middle school leaving certificate	7 (3)	17 (5)	18 (6)	16 (10)
A-levels	66 (30)	24 (7)	167 (57)	6 (4)
Bachelor’s degree	127 (58)	93 (28)	96 (33)	55 (33)
Master’s degree	15 (7)	173 (52)	11 (4)	77 (47)
Doctorate	3 (1)	24 (7)	1 (<1)	11 (7)
More	0	0	0	0
Occupational status				
Full-time job	58 (27)	253 (76)	46 (16)	127 (77)
Part-time job	21 (10)	33 (10)	13 (4)	20 (12)
Job seeker	0	8 (2)	2 (1)	5 (3)
Pensioner	0	1 (<1)	0	2 (1)
Self-employed	0	14 (4)	1	9 (5)
Housewife/househusband	3 (1)	8 (2)	3 (1)	1 (1)
Student	136 (62)	14 (4)	228 (78)	1 (1)
AI usage				
Work	123 (56)	160 (48)	68 (23)	46 (28)
Entertainment	18 (8)	55 (17)	34 (12)	36 (22)
Information purposes	63 (29)	92 (28)	159 (54)	67 (41)
Other	14 (6)	24 (7)	32 (11)	16 (10)
TAM				
Perceived usefulness	25.58 (3.16)	13.82 (4.16)	24.10 (3.03)	12 (4.47)
Perceived ease of use	16.99 (2.70)	13.18 (3.37)	13.12 (4.44)	12.28 (3.72)
Subjective norm	15.41 (2.93)	11.22 (3.51)	13.00 (3.47)	10.2 (3.40)
Attitude toward using	11.32 (3.42)	12.55 (3.45)	10.80 (3.52)	10.97 (3.51)
Behavioral intention	16.48 (2.04)	14.64 (3.07)	13.77 (3.29)	13.40 (3.36)
AIMS				
Non-deskilling	20.23 (3.59)	17.20 (4.77)	16.83 (4.81)	17.87 (5.04)
Growth	17.92 (3.20)	14.12 (4.23)	11.05 (3.79)	12.89 (4.53)
AIMS total score	38.15 (5.10)	31.33 (6.56)	27.88 (6.14)	30.76 (7.02)

Standard deviation in brackets; the percentages have been rounded to whole numbers.

The AI mindset differed significantly among the clusters *F*(3, 1,003) = 116.15, *p* < 0.001. Particularly, the first cluster exhibited a higher AI mindset (*M* = 38.15; *SD* = 5.10) and differed from Cluster 2 (*M* = 31.33; *SD* = 6.56), *t*(531.90) = 13.67, *p* < 0.001, *d* = 1.16; Cluster 3 (*M* = 27.88; *SD* = 6.14), *t*(502.77) = 20.60, *p* < 0.001, *d* = 1.82; and Cluster 4 (*M* = 30.76; *SD* = 7.02), *t*(286.82) = 11.43, *p* < 0.001, *d* = 1.20.

The comparison between genders indicated that male participants showed a slightly higher attitude towards AI (*t*(1002) = 1.96, *p* = 0.05, *d* = 0.12) and perceived ease of use (*t*(1002) = 2.88, *p* = 0.004, *d* = 0.18). No significant gender differences were observed for perceived usefulness (*t*(1002) = 1.36, *p* = 0.17, *d* = 0.09), behavioral intention (*t*(1002) = 1.64, *p* = 0.10, *d* = 0.10), or subjective norm (*t*(1002) = 1.41, *p* = 0.16, *d* = 0.09).

### 4.3 Qualitative analysis

The qualitative analysis of the open-ended responses regarding AI usage revealed that AI is most frequently used for research and idea generation (30% of all open responses). Other common applications of AI included text correction and optimization (13%), text formulation (12%), and coding (9%) (Table 4).

**Table 4:** Qualitative analysis of AI use by clusters.

	Cluster 3	Cluster 2	Cluster 4	Cluster 1	Total
	Early adopter	Early majority	Late majority	Laggard	
Number of answers	36	114	6	39	195 (100)
Text					
Formulate	8	8		8	24 (12)
Review/Correction/Optimization	4	14	2	5	25 (13)
Research/Ideas/ Brainstorming	9	34	2	13	58 (30)
Summarizing	2	8		1	11 (6)
Mailing/Letter/Social Media	2	7		2	11 (6)
Translation	2	7			9 (5)
Images					
Outlines	1				1 (1)
Images editing; generation	1	4	1	1	7 (4)
Tables		2	1		3 (2)
Person or image identification		2			2 (1)
Video editing/ voice overlay		1			1 (1)
Code					
Coding	3	9		5	17 (9)
Debugging	1	3			4 (2)
Optimization	1	1			2 (1)
Data/Analysing		4			4 (2)
Task					
Finance		1		1	2 (1)
Problem solving/SEO	2	3			5 (3)
Automatization/Routines		6		3	9 (5)

Percentages in brackets have been rounded to whole numbers; Cohen’s *d* as effect size for measuring differences between the subsamples.

## 5 Discussion

This study pursued two research objectives. Firstly, we aimed to validate the TAM within the context of AI and extend it to incorporate the Big Five personality traits and the AI mindset. Secondly, our investigation aimed to explore and identify distinct AI adopter types characterized by demographics, AI use, and attitudes toward AI.

### 5.1 The technology acceptance model in the context of AI

The TAM is widely recognized as one of the most utilized and well-validated models for studying technology acceptance. Overall, the study results concerning TAM in the context of AI demonstrated the expected relationships and replicated previous findings in contexts such as VR (Jang et al., 2021), construction software (Park and Park, 2020), or AI applications like ChatGPT (Saif et al., 2024). Specifically, the results indicated that perceived usefulness is the most significant predictor of attitude towards use, which aligns with findings regarding AI usage in online shopping (Hajdú and Nagy, 2021). Perceived ease of use also emerged as a significant predictor of attitude towards use and simultaneously exerted a positive influence on perceived usefulness, consistent with results in the e-commerce sector (Wang et al., 2023). Although perceived ease of use has a lower direct impact on attitude towards use, the substantial influence on perceived usefulness suggests that easily usable, user-friendly technologies are also perceived as useful. In the realm of e-bike sharing, perceived ease of use even proved to be the strongest predictor of attitude towards use (Li et al., 2022). In contrast, Ibrahim et al.'s (see footnote 1) study on VR as a digital interface for military operational command found perceived ease of use not a significant predictor of attitude towards use. Overall, the influence of perceived ease of use and perceived usefulness significantly depends on the context of the technology. In technologies with a clear use case, such as e-bike sharing, perceived ease of use appears to play a greater role, while perceived usefulness seems to be the more important predictor in technologies with higher degrees of freedom and broader horizons of possibilities.

Subjective norm (also social norm) showed only a weak association with attitude towards use. This finding may stem from the fact that the use of AI applications like ChatGPT is minimally influenced by social norms due to their low visibility and therefore low endorsement possibilities by the social environment. Similarly, Mohr and Kühl (2021), investigating AI usage in agriculture, found no effect of subjective norm on attitude towards use. At the same time, Wang et al. (2023) found a positive influence of social norms on perceived ease of use and perceived usefulness, suggesting a model alteration that could be the subject of future investigations. Attitude toward the technology emerged as a strong predictor of behavioral intention to use AI, consistent with numerous studies (Fussell and Truong, 2021; Esteban-Millat et al., 2018).

Another TAM variable not examined in this study was perceived enjoyment (PE). Sohn and Kwon (2020) identified Perceived Enjoyment as the most significant predictor for attitude towards use in their study on technology acceptance in the context of intelligent products. They attributed this strong effect to the shift from innovators to early adopters, who have less technical expertise and interest and place more value on entertainment. However, we did not investigate the factor of PE, assuming that AI chatbots are primarily used as tools. This view is supported by the results of Goli et al. (2023), who also found no influence of PE on behavioral intention in the context of chatbots. Sohn and Kwon (2020) found subjective norm as the second largest predictor in their study, which differs from the results of this investigation, emphasizing the significant influence of the technology context on TAM relationships.

### 5.2 The extended technology acceptance model

The extension of TAM investigated in this study included the AI mindset scales and the Big Five personality dimensions (Costa and McCrae, 2008). The results indicated that non-deskilling had no influence on attitude towards use. According to the findings, the potential fear of lazy delegation and skill loss due to AI usage seems to have no effect on attitudes toward AI usage. However, the AIMS scale growth showed the second strongest effect on attitudes toward AI usage. The potential for personal growth through AI usage thus has a stronger influence on attitude towards use than perceived ease of use or subjective norm. This association suggests that growth plays an important role in AI adoption and technology acceptance. Therefore, the AI mindset appears to be an important interpersonal variable, shaped less by the technical implementation of a platform and more by the individual user’s perspective and expectations.

The investigation of the Big Five showed that the personality trait openness, associated with curiosity and the motivation to gain new knowledge, correlated with perceived ease of use, confirming previous associations (Manis and Choi, 2019; see footnote 1). However, openness did not prove to be a predictor of perceived ease of use, as in the study by Svendsen et al. (2013). The lack of association may be due to the fact that the possibilities of application do not require high levels of openness due to, e.g., ChatGPT’s high visibility and simple interface design. The hypothesis that introverted individuals exhibit higher AI acceptance (Logg et al., 2019) could not be confirmed based on the relationship between extraversion and perceived usefulness. Interestingly, Further, we, indicating low emotional stability, showed no influence on perceived ease of use, although neuroticism is associated with negative emotions when facing change (Terzis et al., 2012) and frustration (Widiger and Oltmanns, 2017). At the same time, neuroticism showed the expected negative relationship with the AIMS scales, supporting previous findings on the relationship between neuroticism and fear of AI (Sindermann et al., 2022). Therefore, neuroticism serves as an indicator of reduced AI mindset and AI adoptability, potentially contributing to the observed gender gap in AI adoption, with women displaying lower adoption rates (Carvajal et al., 2024). Conversely, conscientiousness showed a positive association with non-deskilling, which can be explained by the fact that conscientious individuals have more self-control (Widiger and Oltmanns, 2017), reducing the likelihood of excessive use and lazy delegation. Agreeableness, as a trait indicating greater engagement with interpersonal relations (Graziano and Tobin, 2002), was shown to moderate the effect of subjective norm on behavioral intention (Devaraj et al., 2008). Similarly, the results of this study show that agreeableness is weakly associated with subjective norm. Therefore, individuals with higher agreeableness are likely to adopt AI more readily within environments where AI use is prevalent. AI adoption may occur in waves, with a tipping point in societal adoption potentially triggering a rapid increase in adoption among agreeable individuals who conform to their surroundings.

Another TAM variable not examined in this study was perceived enjoyment (PE). Sohn and Kwon (2020) identified Perceived Enjoyment as the most significant predictor for attitude towards use in their study on technology acceptance in the context of intelligent products. They attributed this strong effect to the shift from innovators to early adopters, who have less technical expertise and interest and place more value on entertainment. However, we did not investigate the factor of PE, assuming that AI chatbots are primarily used as tools. This view is supported by the results of Goli et al. (2023), who also found no influence of PE on behavioral intention in the context of chatbots. Sohn and Kwon (2020) found subjective norm as the second largest predictor in their study, which differs from the results of this investigation, emphasizing the significant influence of the technology context on TAM relationships.

### 5.3 AI adoption types

The exploratory investigation on a typology of AI adopters using k-prototype analysis revealed a four cluster solution as best fitting. For interpreting the clusters, the diffusion of innovations model by Rogers (1962) was used, which has also been validated in the context of AI (Lund et al., 2020; Xu et al., 2023).

The first identified type according to the diffusion of innovations model were the early adopters within the first cluster (*n* = 218; 22%). Early adopters purchase products in an early stage, are less technocratic, and are visionary in discovering applications (Chintalapati, 2021). They play a key role in the technology diffusion process by advising others and increasing the general societal acceptance of a technology (Karnowski and Kümpel, 2016). The subsample of Cluster 1 has a low mean age, a high level of education, and the highest self-reported computer experience. Additionally, this cluster exhibits the highest AI use frequency (several days per week) and consists mostly of students using AI for their work. This group also demonstrates the highest degree of perceived usefulness regarding AI and the highest AI mindset. Cluster 1 comprises individuals who have not only experimented with AI but have firmly integrated the technology into their work processes.

The early majority, according to the diffusion of innovations model, comprises pragmatic technology users who are motivated to try out new technologies by the positive experiences of early adopters (Raman et al., 2023). The early majority is less opinion-leading but well-connected and further promotes the diffusion initiated by the early adopters. Therefore, the transition from the early adopter to the early majority phase, according to Moore (1999), is considered a breakthrough in the diffusion of technology. The second cluster in this study (*n* = 333; 34%) consists of a slightly older, highly educated, full-time working, and predominantly female individuals, with increased computer experience. This group has a moderate AI mindset but relatively low perceived usefulness and perceived ease of use. This group may still be in the experimentation phase and despite their high computer experience, uses AI less frequently than once a week. For this group, training in efficient AI usage and UX design optimizations may be particularly important to highlight practicality and increase perceived usefulness and perceived ease of use.

The group that adopts technologies after the early majority is the late majority, who cautiously consider innovations and only adopt them when social or economic pressure increases (Rogers, 2003, p. 284). The third cluster (*n* = 293; 34%) had the youngest age and mostly consisted of students at the beginning of their studies who predominantly used AI for informational purposes. This cluster exhibited a larger difference between perceived ease of use and perceived usefulness, indicating that this cluster perceives utility as high but considers the application as complicated. Additionally, this cluster showed the lowest AI mindset. Thus, this group may perceive AI usage, especially in education, as a hindrance to the learning process and as a distraction. The perceived difficulty in application could also explain why this cluster predominantly uses AI for research purposes as the considerably most easy use case.

The last group of the diffusion of innovations model are the laggards, who are skeptical about technology, are considered past-oriented, and have low tolerance for uncertainty (Karnowski and Kümpel, 2016). Lund et al. (2020) also refer to them as traditionalists. According to this study, the laggards correspond to the fourth cluster (*n* = 165; 16%), with the highest average age, a predominantly male gender, a moderate to low computer experience and the less frequent AI usage (slightly more than once a month). This group is mostly employed full-time and predominantly uses AI for informational purposes. Perceived usefulness and perceived ease of use are the lowest in this cluster. The AI mindset is moderate to low. Despite the high education of this cluster (47% with Master’s degree; 7% with PhD), this group seems to engage less with AI, and their social environment the subjective norm motivates them less to actively engage with AI. According to Chintalapati (2021), challenges in persuading laggards to adopt AI include data privacy and regulatory guidelines, as well as the replace-augment argument, leading to fear of possible job loss or perceived pressure for necessary adaptation. Due to the low perceived usefulness, this group could be motivated to engage with the new technology, especially through legal regulations, and demonstrations illustrating automation possibilities. Particularly if this group consists of executives, their resistance could be crucial for organizational change and implementation of AI in work processes.

Overall, this exploratory study demonstrates, on the one hand, that Rogers (1962) diffusion of innovations model is applicable to AI adoption. Also, the comparison of the percentage shares of the individual groups described by Rogers (2003) (early adopters = 13.5%; early majority = 34%; late majority = 23%; and laggards = 16%) appears comparable in this study (22%; 33%; 29%; 16%), with the group of early adopters being larger and the late majority being smaller. The number of individuals in each cluster shows that AI has already diffused into society, and the breakthrough, transitioning from early adopters to the early majority, has occurred. However, the qualitative study shows that AI is predominantly used for research and informational purposes, text correction, and simple formulations. Therefore, the potential of AI for automating work processes is not yet fully realized and will increasingly become important for economic and scientific progress in the future.

### 5.4 Implications

The results indicate that AI chatbots like ChatGPT are primarily utilized due to their utility and primarily serve as tools, suggesting that the development focus should prioritize perceived usefulness and perceived ease of use. This sets AI chatbots apart from intelligent products, where perceived enjoyment primarily influences attitude towards use (Sohn and Kwon, 2020).

The subjective norm has little influence on attitudes toward AI chatbots, suggesting that products like ChatGPT have become mainstream and widely accepted in society. However, concerns regarding data misuse persist in both the business and societal contexts (Huang et al., 2023), indicating that increased transparency from AI application providers could further enhance attitudes and behavioral intentions toward AI, especially among the laggards.

Furthermore, the AI mindset could be a significant lever to further enhance attitudes toward AI and motivation for its use. Interventions could demonstrate how AI can support one’s learning process, act as a mentor in problem-solving, and free up time for learning opportunities by delegating routine tasks. Future research should examine potential interventions using longitudinal designs to explore applications for educational and organizational contexts, which could have a notable impact, particularly among the late majority. Furthermore, policymakers and educational institutions should address gender differences in AI mindset and adoption (Carvajal et al., 2024) to foster gender equality in the future labor market.

To enhance the practical relevance of these findings for organizations and policymakers, we provide targeted recommendations based on cluster insights. For *early adopters*, strategies could focus on reinforcing engagement by providing advanced AI features and opportunities for skill development, as these individuals are likely to act as advocates and role models. For the *early majority*, organizations might emphasize practical, productivity-focused applications of AI, accompanied by training that underscores ease of use, to transition them from experimentation to regular use. The *late majority* may benefit from peer-led initiatives and clear, practical demonstrations of AI’s utility to reduce perceived complexity and increase perceived usefulness. Lastly, for *laggards*, targeted communication addressing privacy concerns and job security, along with regulatory incentives, may help alleviate resistance to AI adoption.

Subsequent studies could investigate interventions aimed at increasing the AI mindset using longitudinal designs and their effects on actual AI use. However, the rapid development of AI applications necessitates ongoing model adaptations. For instance, the recent rollout of OpenAI GPT-4o, a model capable of reasoning across audio, video, and text in real-time, demonstrates remarkable capabilities, opening avenues for further technology acceptance research due to its new interaction possibilities. It is conceivable that perceived enjoyment then will become a significant factor (see Sohn and Kwon, 2020). Given its significantly anthropomorphized model with emotional vocal expression, investigations into an AI uncanny valley could also be a promising future research direction.

### 5.5 Limitations

This study has several limitations that warrant discussion. Firstly, the sample examined in this study consisted predominantly of individuals with bachelor’s or master’s degrees, indicating above-average levels of education. Additionally, only individuals with at least one AI experience were eligible to participate in the study, further limiting the representativeness of the results. Therefore, especially the laggard adopter type may not be fully representative. To obtain a more accurate depiction, future research should include individuals without direct experience with AI applications, particularly to better understand the characteristics of the laggards. Furthermore, the study exclusively examined the German-speaking population. Investigations in other cultures are necessary to evaluate the results independently of cultural influences. Additionally, cross-cultural equivalence of constructs such as the AI mindset could be an important future research topic.

## 6 Conclusion

The use of AI chatbots appears to have transitioned from the phase of evaluation and consideration, which is more strongly influenced by social norms, to the phase of acceptance and adoption. This assumption is supported by the exploratory k-prototype analysis, where the early majority represented the largest group. It is evident that perceived usefulness, in particular, plays a crucial role in shaping attitudes toward a technology. Furthermore, the AIMS scale growth emerged as the second-largest predictor of attitude toward use, suggesting the potential role of the AI mindset in encouraging individuals to experiment with AI. The results indicate that AI applications such as ChatGPT have become mainstream, highlighting the importance of research on human-AI interaction. Understanding the utility and simplicity of these applications through UX design and use cases, as well as comprehending people’s attitudes and concerns regarding AI, are crucial steps toward making the technology accessible and beneficial to all sectors of society.

## Data Availability

The datasets presented in this study can be found in online repositories. The names of the repository/repositories and accession number(s) can be found in the article/Supplementary material.

## Footnotes

^1^Ibrahim, F., Schumacher, J., Hofer, P., and Daseking, M. (n.d.-a). Decision-making in virtual reality: An experimental study on virtual reality’s effect on presence, task-load, and decision-making performance within operational command (manuscript under review).

^2^Ibrahim, F., Telle, N. T., Herzberg, P. Y., and Müncher, J. C. (n.d.-b). The construction and validation of the AI mindset scale (AIMS): Personality Psychology and Psychological Assessment: Helmut-Schmidt-University.
